# Progress Towards Mammalian Whole-Brain Cellular Connectomics

**DOI:** 10.3389/fnana.2016.00062

**Published:** 2016-06-30

**Authors:** Shawn Mikula

**Affiliations:** Max-Planck Institute for Neurobiology, Electrons - Photons - NeuronsMartinsried, Germany

**Keywords:** whole-brain, sample preparation, ultramicrotomy, electron microscopy, connectomics

## Abstract

Neurons are the fundamental structural units of the nervous system—i.e., the Neuron Doctrine—as the pioneering work of Santiago Ramón y Cajal in the 1880’s clearly demonstrated through careful observation of Golgi-stained neuronal morphologies. However, at that time sample preparation, imaging methods and computational tools were either nonexistent or insufficiently developed to permit the precise mapping of an entire brain with all of its neurons and their connections. Some measure of the “mesoscopic” connectional organization of the mammalian brain has been obtained over the past decade by alignment of sparse subsets of labeled neurons onto a reference atlas or via MRI-based diffusion tensor imaging. Neither method, however, provides data on the complete connectivity of all neurons comprising an individual brain. Fortunately, whole-brain cellular connectomics now appears within reach due to recent advances in whole-brain sample preparation and high-throughput electron microscopy (EM), though substantial obstacles remain with respect to large volume electron microscopic acquisitions and automated neurite reconstructions. This perspective examines the current status and problems associated with generating a mammalian whole-brain cellular connectome and argues that the time is right to launch a concerted connectomic attack on a small mammalian whole-brain.

## Introduction

Perception, cognition and behavior are consequences of neural computations executed by neuronal circuits that are widely distributed throughout the whole-brain. Since its inception, neuroscience has attempted to interpret brain organization and function without a detailed map of its total neuronal constituents and their complete inter-connectivities at the required synaptic resolution. Without such a map, a whole-brain cellular connectome, it is highly improbable that the precise neural computations underlying brain and behavior will ever be adequately understood. Currently only electron microscopy (EM) provides the resolution necessary to reliably reconstruct all neuronal circuits contained within a given volume in terms of individual synapses, though continued improvements in super-resolution light microscopy may one day provide a suitable alternative. A comprehensive reconstruction of a whole-brain synaptic “wiring diagram” based on high-throughput EM is thus highly desirable.

Several reviews covering cellular connectomics have appeared in recent years (Lichtman and Sanes, 2008; Lichtman and Denk, 2011; Denk et al., 2012; Helmstaedter, 2013; Peddie and Collinson, 2014; Wanner et al., 2015). A discussion of the progress and problems specifically associated with whole-brain cellular connectomics has not yet appeared. Here I discuss the concrete steps that can lead to a cellular connectome of a small mammalian whole-brain.

## The Critical Steps

**Figure 1:**
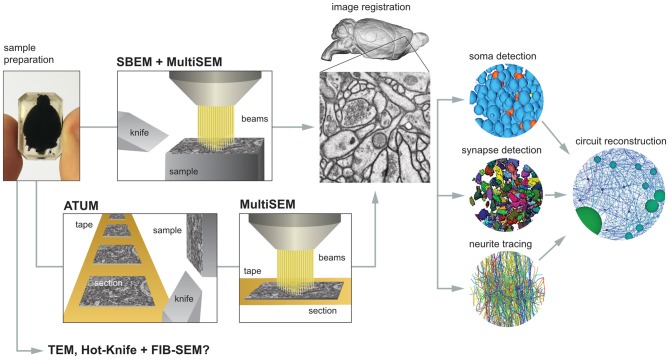
**The critical steps to mammalian whole-brain cellular connectomics.** Shown are the main steps and their dependencies for reconstructing a mammalian whole-brain circuit: sample preparation, serial block-face electron microscopy (SBEM), automated tape collecting ultramicrotomy (ATUM), serial section multiSEM imaging, image registration, soma detection, synapse detection, neurite tracing and circuit reconstruction. See text for details.

There are a number of steps necessary for reconstructing the mammalian whole-brain neural circuit *in toto* (Figure 1).

### Sample Preparation

The first step in the process is the sample preparation. Perfusions of whole-brains with osmium tetroxide solutions were performed in the 1960’s but were not entirely successful as white matter was not completely stained, nor was whole-brain plastic embedding employed (Palay et al., 1962). Recently, whole mouse brain staining and embedding employing diffusion was described. The BROPA method (Brain-wide formamide-Reduced-Osmium staining with Pyrogallol-mediated osmium Amplification) results in the preservation and staining of the cellular ultrastructure throughout the brain at a resolution sufficient for reliably tracing neurites and identifying synapses, which are both necessary and sufficient for the reconstruction of brain-wide neuronal circuits (Mikula and Denk, 2015). Further improvements of the BROPA method and its application to larger species (e.g., rat, marmoset and macaque) are currently underway.

X-ray micro-computed tomography (i.e., X-ray microCT) is one method for non-invasively imaging whole brains at the micro-scale, which is suitable for assessing brain integrity (e.g., detection of sample cracks and dissection-related damage) and stain uniformity. X-ray microCT relies on the detection of transmitted X-rays (i.e., projections) through a thick sample. A series of X-ray projections from a range of angles is then used for tomographic reconstruction by converting image data in the Radon transform domain to a volume dataset in the spatial domain (Paulus et al., 2000). Since the contrast obtained from X-ray microCT is similar to that of volume EM (Mikula and Denk, 2015; Dyer et al., 2016), it may be considered a downsampled version of the volume EM dataset. Typical resolutions from commercial X-ray microCT systems range from 10 to 15 microns for small mammalian whole-brain samples prepared for EM. The use of brighter, coherent light sources (e.g., synchotron radiation) can improve resolution in these samples down to one micron or possibly better (Dyer et al., 2016), though it is currently unclear whether these resolution gains will be sufficient for mammalian whole-brain cellular connectomics.

**Figure 2:**
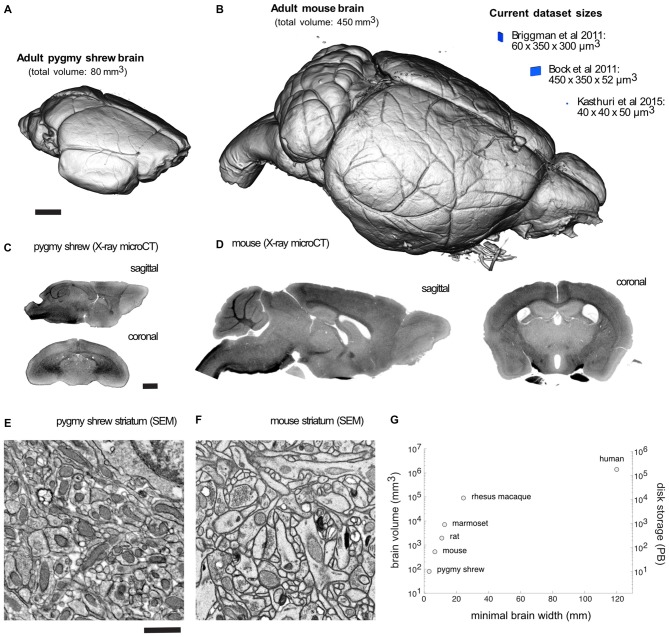
**Mammalian whole-brain sample preparation for electron microscopy (EM).** Volume visualization of an X-ray microCT dataset of an adult **(A)** pygmy shrew and **(B)** mouse brain prepared for EM using a Brain-wide formamide-reduced-osmium staining with pyrogallol-mediated osmium amplification (BROPA)-like protocol (Mikula and Denk, 2015). Recent EM dataset volumes (blue), all less than 0.01 mm^3^, are shown adjacent to the mouse brain for comparison. Sagittal and coronal slices through an X-ray microCT dataset for the **(C)** pygmy shrew and **(D)** mouse brain. Scanning electron micrographs from the striatum of the **(E)** pygmy shrew and **(F)** mouse brain shown in **(A,B)**, respectively. **(G)** Scatter plot of mammalian brain volumes, disk storage requirements, assuming 20 nm isotropic voxel size, and minimal brain width, which is important for diamond knife cutting requirements. Imaging parameters in **(E,F)** are similar to those from Mikula and Denk, 2015. X-ray microCT and EM image intensities are inverted. Scale bars are 1 mm in **(A,C)**, and 1 μm in **(E).** Scale bar in **(A)** applies to **(B), (C)** applies to **(D), (E)** applies to **(F)**.

Volume visualization of an X-ray microCT dataset of an adult Etruscan pygmy shrew and mouse brain prepared for EM demonstrates completely intact brains (Figures 2A,B). Recent EM dataset volumes (blue) from Briggman et al. (2011), Bock et al. (2011), and Kasthuri et al. (2015), are shown adjacent to the mouse brain for comparison. Sagittal and coronal slices through an X-ray microCT dataset for the pygmy shrew and mouse brain confirm the absence of cracks and complete, uniform staining (Figures 2C,D). Scanning electron microscopy (SEM) of the striatum of the pygmy shrew and mouse brain (Figures 2E,F) demonstrates ultrastructural preservation and high membrane contrast that, in the mouse where it has been quantified, appears sufficient for reconstructing even the finest neuronal processes and synapses (Mikula and Denk, 2015).

### Volume EM

There are two routes available for high-throughput, large-volume EM, using either serial block-face electron microscopy (SBEM, Figure 1; Denk and Horstmann, 2004; Briggman et al., 2011) or automated tape-collecting ultramicrotomy (ATUM; Hayworth et al., 2006; Schalek et al., 2011). In SBEM, an SEM in-chamber ultramicrotome sequentially removes ultrathin sections from the sample, exposing a new surface (i.e., block-face) on the sample, which is then imaged by SEM. The removed sections are neither collected nor imaged and can pose a debris problem in the SEM if not properly handled. In ATUM, serial sections are produced on a standard ultramicrotome and collected automatically on tape, which is subsequently imaged via SEM.

The primary technical challenge for whole-brain SBEM is the design of the in-chamber microtome and nano-positioning machines with sufficient precision and stiffness, whereas the main challenge using ATUM involves the reliable collection of thin, intact, wrinkle- and chatter-free sections. To image a whole-brain using a conventional single-beam microscope is impractical; thus both volume EM imaging routes critically depend on multi-beam SEM (multi-SEM), a recent advance in imaging technology (Eberle et al., 2015; Kemen et al., 2015), to achieve the throughput (i.e., >1 GHz image acquisition rate, inclusive of stage movement overhead) necessary for imaging mammalian whole-brains within an acceptable period of time at a resolution sufficient for reconstructing neuronal circuits (e.g., 20 nm, isotropic).

Additional potential routes for mammalian whole-brain volume EM cannot be ruled out (Figure 1). For example, TEM (Bock et al., 2011; Marc et al., 2013) or combined Hot-Knife and FIB-SEM (Hayworth et al., 2015) may allow for parallel imaging, though in place of multiple e-beams, multiple TEMs or FIB-SEMs are required. However, due to the slower imaging throughputs, 5–8 MHz for TEM (Bock et al., 2011; Lee et al., 2016) and 1 MHz for FIB-SEM (Hayworth et al., 2015), roughly 100–200 TEMs or 1000 FIB-SEMs would be required to match the imaging throughput of a single multiSEM, which can exceed 1 GHz (Kemen et al., 2015), making TEM or FIB-SEM approaches apparently less cost effective and practical.

Volume EM of the smallest adult mammalian brains, Etruscan pygmy shrew or bumblebee bat, with an estimated brain volume of approximately 80 mm^3^, requires about 3 months, whereas the adult mouse brain at 450 mm^3^ requires 1.5 years, using a 1 GHz image acquisition rate and 20 nm voxel size. In principle, imaging times can be substantially reduced by the addition of more beams to the multi-SEM, allowing for more than an order of magnitude reduction in imaging times, thus providing a viable solution to imaging larger brains (e.g., rat, marmoset, even human).

### Data Storage

The decision to store whole-brain volume EM data on spinning disks or tape media will depend largely on the trade-off between data access rates vs. storage capacity cost, though additional factors such as reliability, security and mobility may be considered. For the smallest mammalian brains (i.e., Etruscan pygmy shrew or bumblebee bat), the cost for the 10 PB storage required (Figure 2G), imaged at 20 nm isotropic voxel, is about 100 k Euros for tape storage vs. 500 k Euros for disk, assuming current estimates of 0.01 Euro/GB for tape storage and 0.05 Euro/GB for disk. For a mouse whole-brain, storage requirements increase to 56 PB and tape storage costs to 560 k Euros (or 2.8 million Euros for disk), which are well within current institutional capabilities and funding. For the human brain, however, the expected 175 Exabytes storage requirement (Figure 2G), costing 1.75 billion Euros for tape (or 8.75 billion Euros for disk), will likely exceed individual institutions, thus requiring collective efforts.

### Image Registration

The assembly of 2D images into a registered 3D volume is a largely solved problem and several acceptable solutions exist (Guest and Baldock, 1995; Saalfeld et al., 2010; Tasdizen et al., 2010). In the case of SBEM, the registration problem is substantially simpler due to the general absence of nonlinear distortions in the images, requiring only the estimation of image translational offsets determined using a similarity measure such as cross-correlation that is subsequently used to obtain a global least squares solution of the linear system of image offset equations (Denk and Horstmann, 2004; Briggman et al., 2011; Mikula et al., 2012). In addition, the use of X-ray microCT whole-brain data as 3D “ground truth” is expected to substantially facilitate the EM image registration.

### Image Post-Processing and Circuit Reconstruction

There are three steps that can begin in parallel once registered volume EM data becomes available. For soma and synapse detection, reliable methods have been developed that generally involve classification of image feature vectors with additional post-processing (Kreshuk et al., 2011; Morales et al., 2011; Becker et al., 2013; Tek et al., 2014; Márquez Neila et al., 2016). For neurite tracing or segmentation, though progress has been made (Jain et al., 2010; Kaynig et al., 2013; Liu et al., 2014; Perez et al., 2014; Plaza et al., 2014; Berning et al., 2015; Ai-Awami et al., 2016) the problem remains unsolved. Crowd-sourcing efforts that rely on manual neurite tracing (Arganda-Carreras et al., 2015) are currently used for annotating small-volume EM datasets (see Figure 2B, Current dataset sizes) but will not scale to larger volumes such as small mammalian whole-brains. For example, given the estimate of 5 h/mm manual neurite tracing speed from Berning et al. (2015), it would take more than 500,000 years to manually trace all neurites in the mouse brain (assuming 100 million neurons with an average neurite length per neuron of 10 mm) and 50 billion Euros (assuming 10 Euros/h). Fortunately, there are alternatives on the horizon. Whether the solution comes from deep machine learning methods currently being used in SEM (Huang and Jain, 2013), from autotracing methods originally developed for light microscopy (Ming et al., 2013) and applied to cellular boundary probability maps, or from elsewhere remains to be seen.

Can image processing demands keep pace with imaging acquisition throughput? This is a complicated question due to the multiple possible post-processing strategies that can be deployed. For example, it is likely that distributed image registration and reliable nuclei and synapse detection can be performed before image data is written to disk or tape, where subsequent retrieval would prove costly in terms of image read times. If this is not possible and image data is written to disk or tape before registration and additional post-processing, then subsequent computations requisite for registration and segmentation may prove to be a bottleneck. In any event, the large petabyte-scale EM volumes characteristic of small mammalian whole-brains are not expected to pose an insurmountable computer processing problem as block-based segmentations of large EM datasets have recently been demonstrated (Kroeger et al., 2013) and it is expected that such block-based approaches, in conjunction with CPU-GPU clusters for high-performance computing, will accommodate even mammalian whole-brain EM datasets.

Once somas and synapses are autodetected and neurites are autotraced, the final step of neuronal circuit reconstruction involves assigning the detected synapses to pre- and post-synaptic neurites, assigning neurites to connecting somas and by the transitive relation, connecting synapses to their pre- and post-synaptic somas.

## Real and Potential Issues

### General Technical Considerations

There are a number of problems that are expected with the approach described here besides the need for algorithms capable of reliable neurite autotracing as discussed in the preceding section. The long duration of the volume EM acquisitions will pose a challenge. In the case of the mouse brain, the estimated 1.5 years required for volume EM data acquisition is an order of magnitude longer than current volume EM acquisitions (Bock et al., 2011; Briggman et al., 2011), which necessitates the ability to pause the acquisition as soon as a problem, such as component failure, is detected, correct the problem and then restart the acquisition without any data loss. The complexity of the multiSEM and nanopositioning machines will make this a challenge as there are many components that can fail or malfunction. Unintended diamond knife damage and likely inevitable dulling, both requiring knife replacement, are problems that may prove difficult to overcome if hundreds of thousands of consecutive thin sectionings are required. In this regard, non-mechanical approaches to removing material, such as ion milling, offer a distinct advantage, though their suitability to multiSEM secondary electron (SE) imaging has not yet been demonstrated and may be problematic due to ion beam-induced surface streaking. In addition, since EM necessarily involves irradiating a sample with electrons (typically at high currents, such as nA), sample charging is a common problem in high-vacuum conditions, resulting in information loss and image aberrations, though using in-chamber evaporative coating with metals (Titze and Denk, 2013) or including or synthesizing *en bloc* conductive polymers can address this problem.

### Validation of Circuit Reconstructions

An important issue is validation of the reconstructed brain-wide circuits. While consensus of redundantly- and manually-traced neurites and annotated synapses is one approach (Helmstaedter et al., 2011; Mikula and Denk, 2015), it relies on model-based assumptions. A more convincing route may involve comparison of EM-based circuit reconstructions with light microscopic reconstructions of sparse labeling in the same animal, such as may be obtained with monosynaptic deletion-mutant rabies virus (Wickersham et al., 2006). Another route may involve imaging a sub-volume at a higher resolution, such as 2–5 nm, in order to assess whether all small synapses and fine neurites can be reliably detected and annotated throughout the full volume acquired at a coarser resolution of 10–20 nm. Although recent results suggest that 10 nm in-plane resolution and 30 nm slice thickness are sufficient for reliable circuit reconstructions in whole mouse brain preparations (Mikula and Denk, 2015), further validation of these results in different areas of the brain is prudent. It should be noted that different sample preparations and volume imaging methods are expected to have different resolution requirements for circuit reconstruction (e.g., Mishchenko et al., 2010).

### Missing Neurochemistry and Gap Junctions

To what extent will a whole-brain cellular connectome obtained from EM permit inferences about neuronal function? Do we need detailed neurochemical information or is ultrastructure in addition to known cell-type neurochemical information sufficient? Certainly the existence of silent synapses (Atwood and Wojtowicz, 1999), gap junctions, more than 50 chemically-distinct neurotransmitters and myriad receptor subtypes with widely-varying post-synaptic responses would urge caution (see Bargmann and Marder, 2013).

Gap junctions deserve special emphasis since they are prevalent in mammalian brains (Rozental et al., 2000) and at least for *C. elegans*, form networks that are not correlated with synaptic networks (Varshney et al., 2011). Unfortunately, gap junctions are generally difficult to reliably identify in conventional EM preparations (Varshney et al., 2011) and may necessitate the use of modified sample preparations for enhanced gap junction staining (van Deurs, 1975; Baker et al., 1985) or the preservation of the extracellular space, which provides indirect evidence for their presence (Cragg, 1980; Pallotto et al., 2015).

It must be emphasized that ultrastructural maps of whole-brain synaptic connectivity likely do not contain all requisite information for accurate whole-brain simulations but we will not know for certain what additional neurochemical information is needed until we are in possession of such connectional maps from mammalian whole-brains, the lessons of the *C. elegans* nervous system and crustacean stomatogastric ganglia notwithstanding. Additional neurochemical information will likely necessitate the development of novel, high-throughput imaging methods beyond the scope of the ultrastructural approach described here. Nonetheless, we may ask, how far can mammalian whole-brain ultrastructure inform us about function and behavior? We are now in a position to find out.

## Prospective

### Realising Cajal’s Vision

The Neuron Doctrine, established in the 1880’s (Ramón y Cajal, 1889), has been central to our understanding of neuronal circuit organization and has lead to inferences over neuronal function; e.g., direction of information flow in the hippocampal circuit (Ramón y Cajal, 1995). The methods in Ramon y Cajal’s time were clearly inadequate to map the connections between all neurons in a mammalian whole-brain, but today we are in possession of the tools required to realize this vision, bringing the neuroanatomical program he initiated to completion.

### What will a Mammalian Whole-Brain Circuit Diagram Tell Us?

The only nervous system whose circuitry has been completely mapped is that of the worm, *C. elegans* (White et al., 1986; Emmons, 2015). From this precedent, we can be confident that a mammalian whole-brain neuronal connectivity map will not only inspire and guide countless neuroscience investigations but will also contribute to a better understanding of the neuronal basis of brain, behavior and ultimately, ourselves. But what, specifically, will a mammalian whole-brain circuit diagram tell us? Besides indicating pathways for information flow and the possibility of testing competing neuronal computational models, a complete mammalian whole-brain circuit map will allow us to revisit the concept of the “brain area”, an abstraction whose usefulness in early sensori-motor processing stages is generally accepted due to the topographic mapping of sensory receptors and motor effectors, but whose utility in higher stages of processing is far from clear. Historically, architectonic differentiation, cytochemical signatures and physiological properties have been used to demarcate brain areas (Felleman and Van Essen, 1991), though these approaches are vulnerable to subjective bias (but see Schleicher et al., 1999). More recently, spatial clustering of gene expression patterns has been used (Hawrylycz et al., 2011), though the clusters generally demarcate cortical lamina and not areal boundaries. A mammalian whole-brain circuit diagram will allow us to revisit the brain area concept by providing a precise connectional basis for potential brain parcellations and abstractions and is expected to allow us to see the brain, not as a collection of discrete brain areas or cell types with interconnections, but as a complicated network of individual neurons that may defy simplification.

## Author Contributions

SM performed the experiments and analysis and wrote the article.

## Funding

Supported by the Max Planck Society.

## Conflict of Interest Statement

The author declares that the research was conducted in the absence of any commercial or financial relationships that could be construed as a potential conflict of interest.

## References

[B1] Ai-Awami A. K. Beyer J. Haehn D. Kasthuri N. Lichtman J. W. Pfister H.. (2016). Neuroblocks—visual tracking of segmentation and proofreading for large connectomics projects. IEEE Trans. Vis. Comput. Graph. 22, 738–746. 10.1109/TVCG.2015.2467441 26529725

[B2] Arganda-Carreras I. Turaga S. C. Berger D. R. Cireşan D. Giusti A. Gambardella L. M.. (2015). Crowdsourcing the creation of image segmentation algorithms for connectomics. Front. Neuroanat. 9:142. 10.3389/fnana.2015.00142 26594156 PMC4633678

[B3] Atwood H. L. Wojtowicz J. M. (1999). Silent synapses in neural plasticity: current evidence. Learn. Mem. 6, 542–571. 10.1101/lm.6.6.542 10641762

[B4] Baker T. S. Sosinsky G. E. Caspar D. L. Gall C. Goodenough D. A. (1985). Gap junction structures. VII. Analysis of connexon images obtained with cationic and anionic negative stains. J. Mol. Biol. 184, 81–98. 10.1016/0022-2836(85)90045-2 2411939 PMC4147872

[B5] Bargmann C. I. Marder E. (2013). From the connectome to brain function. Nat. Methods 10, 483–490. 10.1038/nmeth.2451 23866325

[B6] Becker C. Ali K. Knott G. Fua P. (2013). Learning context cues for synapse segmentation. IEEE Transactions on Medical Imaging, 32, 1864–1877. 10.1109/TMI.2013.2267747 23771317

[B7] Berning M. Boergens K. M. Helmstaedter M. (2015). SegEM: efficient image analysis for high-resolution connectomics. Neuron 87, 1193–1206. 10.1016/j.neuron.2015.09.003 26402603

[B8] Bock D. D. Lee W.-C. A. Kerlin A. M. Andermann M. L. Hood G. Wetzel A. W.. (2011). Network anatomy and *in vivo* physiology of visual cortical neurons. Nature 471, 177–182. 10.1038/nature09802 21390124 PMC3095821

[B9] Briggman K. L. Helmstaedter M. Denk W. (2011). Wiring specificity in the direction-selectivity circuit of the retina. Nature 471, 183–188. 10.1038/nature09818 21390125

[B10] Cragg B. (1980). Preservation of extracellular space during fixation of the brain for electron microscopy. Tissue Cell 12, 63–72. 10.1016/0040-8166(80)90052-x 6987773

[B12] Denk W. Briggman K. L. Helmstaedter M. (2012). Structural neurobiology: missing link to a mechanistic understanding of neural computation. Nat. Rev. Neurosci. 13, 351–358. 10.1038/nrn3169 22353782

[B11] Denk W. Horstmann H. (2004). Serial block-face scanning electron microscopy to reconstruct three-dimensional tissue nanostructure. PLoS Biol. 2:e329. 10.1371/journal.pbio.0020329 15514700 PMC524270

[B13] Dyer E. L. Roncal W. G. Fernandes H. L. Gürsoy D. Xiao X. Vogelstein J. T. (2016). Quantifying mesoscale neuroanatomy using X-ray microtomography. arXiv Preprint arXiv:1604.03629. Retrieved from: http://arxiv.org/abs/1604.03629 10.1523/ENEURO.0195-17.2017 PMC5659258 29085899

[B14] Eberle A. L. Mikula S. Schalek R. Lichtman J. W. Knothe Tate M. L. Zeidler D. (2015). High-resolution, high-throughput imaging with a multibeam scanning electron microscope. J. Microsc. 259, 114–120. 10.1111/jmi.12224 25627873 PMC4670696

[B15] Emmons S. W. (2015). The beginning of connectomics: a commentary on White et al.(1986) “The structure of the nervous system of the nematode *Caenorhabditis elegans*”. Philos. Trans. R. Soc. Lond. B Biol. Sci. 370:20140309. 10.1098/rstb.2014.0309 25750233 PMC4360118

[B16] Felleman D. J. Van Essen D. C. (1991). Distributed hierarchical processing in the primate cerebral cortex. Cereb. Cortex 1, 1–47. 10.1093/cercor/1.1.1 1822724

[B17] Guest E. Baldock R. (1995). Automatic reconstruction of serial sections using the finite element method. Bioimaging 3, 154–167. 10.1002/1361-6374(199512)3:4<154::aid-bio2>3.3.co;2-d

[B18] Hawrylycz M. Ng L. Page D. Morris J. Lau C. Faber S.. (2011). Multi-scale correlation structure of gene expression in the brain. Neural Netw. 24, 933–942. 10.1016/j.neunet.2011.06.012 21764550

[B20] Hayworth K. Kasthuri N. Schalek R. Lichtman J. (2006). Automating the collection of ultrathin serial sections for large volume TEM reconstructions. Microsc. Microanal. 12, 86–87. 10.1017/s1431927606066268

[B19] Hayworth K. J. Xu C. S. Lu Z. Knott G. W. Fetter R. D. Tapia J. C.. (2015). Ultrastructurally smooth thick partitioning and volume stitching for large-scale connectomics. Nat. Methods 12, 319–322. 10.1038/nmeth.3292 25686390 PMC4382383

[B21] Helmstaedter M. (2013). Cellular-resolution connectomics: challenges of dense neural circuit reconstruction. Nat. Methods 10, 501–507. 10.1038/nmeth.2476 23722209

[B22] Helmstaedter M. Briggman K. L. Denk W. (2011). High-accuracy neurite reconstruction for high-throughput neuroanatomy. Nat. Neurosci. 14, 1081–1088. 10.1038/nn.2868 21743472

[B23] Huang G. B. Jain V. (2013). Deep and wide multiscale recursive networks for robust image labeling. arXiv Preprint arXiv:1310.0354. Retrieved from: http://arxiv.org/abs/1310.0354.

[B24] Jain V. Seung H. S. Turaga S. C. (2010). Machines that learn to segment images: a crucial technology for connectomics. Curr. Opin. Neurobiol. 20, 653–666. 10.1016/j.conb.2010.07.004 20801638 PMC2975605

[B25] Kasthuri N. Hayworth K. J. Berger D. R. Schalek R. L. Conchello J. A. Knowles-Barley S.. (2015). Saturated reconstruction of a volume of neocortex. Cell 162, 648–661. 10.1016/j.cell.2015.06.054 26232230

[B26] Kaynig V. Vazquez-Reina A. Knowles-Barley S. Roberts M. Jones T. R. Kasthuri N. (2013). Large-scale automatic reconstruction of neuronal processes from electron microscopy images. arXiv: 1303.7186 7186 [cs, Q-Bio]. Retrieved from: http://arxiv.org/abs/1303.7186. 10.1016/j.media.2015.02.001 PMC4406409 25791436

[B27] Kemen T. Malloy M. Thiel B. Mikula S. Denk W. Dellemann G. (2015). “Further advancing the throughput of a multibeam SEM,” in SPIE Proceedings 9424, Metrology, Inspection, and Process Control for Microlithography (94241U-94241U-6) (San Jose, CA).

[B28] Kreshuk A. Straehle C. N. Sommer C. Koethe U. Cantoni M. Knott G.. (2011). Automated detection and segmentation of synaptic contacts in nearly isotropic serial electron microscopy images. PLoS One 6:e24899. 10.1371/journal.pone.0024899 22031814 PMC3198725

[B29] Kroeger T. Mikula S. Denk W. Koethe U. Hamprecht F. A. (2013). Learning to segment neurons with non-local quality measures. Med. Image Comput. Comput. Assist. Interv. 16, 419–427. 10.1007/978-3-642-40763-5_52 24579168

[B30] Lee W.-C. A. Bonin V. Reed M. Graham B. J. Hood G. Glattfelder K.. (2016). Anatomy and function of an excitatory network in the visual cortex. Nature 532, 370–374. 10.1038/nature17192 27018655 PMC4844839

[B31] Lichtman J. W. Denk W. (2011). The big and the small: challenges of imaging the brain’s circuits. Science 334, 618–623. 10.1126/science.1209168 22053041

[B32] Lichtman J. W. Sanes J. R. (2008). Ome sweet ome: what can the genome tell us about the connectome? Curr. Opin. Neurobiol. 18, 346–353. 10.1016/j.conb.2008.08.010 18801435 PMC2735215

[B33] Liu T. Jones C. Seyedhosseini M. Tasdizen T. (2014). A modular hierarchical approach to 3D electron microscopy image segmentation. J. Neurosci. Methods 226, 88–102. 10.1016/j.jneumeth.2014.01.022 24491638 PMC3970427

[B34] Marc R. E. Jones B. W. Watt C. B. Anderson J. R. Sigulinsky C. Lauritzen S. (2013). Retinal connectomics: towards complete, accurate networks. Prog. Retin. Eye Res. 37, 141–162. 10.1016/j.preteyeres.2013.08.002 24016532 PMC4045117

[B40] Márquez Neila P. Baumela L. González-Soriano J. Rodríguez J.-R. DeFelipe J. Merchán-Pérez Á. (2016). A fast method for the segmentation of synaptic junctions and mitochondria in serial electron microscopic images of the brain. Neuroinformatics 14, 235–350. 10.1007/s12021-015-9288-z 26780198 PMC4823374

[B36] Mikula S. Binding J. Denk W. (2012). Staining and embedding the whole mouse brain for electron microscopy. Nat. Methods 9, 1198–1201. 10.1038/nmeth.2213 23085613

[B35] Mikula S. Denk W. (2015). High-resolution whole-brain staining for electron microscopic circuit reconstruction. Nat. Methods 12, 541–546. 10.1038/nmeth.3361 25867849

[B37] Ming X. Li A. Wu J. Yan C. Ding W. Gong H.. (2013). Rapid reconstruction of 3D neuronal morphology from light microscopy images with augmented rayburst sampling. PLoS One 8:e84557. 10.1371/journal.pone.0084557 24391966 PMC3877282

[B38] Mishchenko Y. Hu T. Spacek J. Mendenhall J. Harris K. M. Chklovskii D. B. (2010). Ultrastructural analysis of hippocampal neuropil from the connectomics perspective. Neuron 67, 1009–1020. 10.1016/j.neuron.2010.08.014 20869597 PMC3215280

[B39] Morales J. Alonso-Nanclares L. Rodríguez J.-R. Defelipe J. Rodríguez Á. Merchán-Pérez A. (2011). ESPINA: a tool for the automated segmentation and counting of synapses in large stacks of electron microscopy images. Front. Neuroanat. 5:18. 10.3389/fnana.2011.00018 21633491 PMC3099746

[B41] Palay S. L. McGee-Russell S. M. Gordon S. Jr. Grillo M. A. (1962). Fixation of neural tissues for electron microscopy by perfusion with solutions of osmium tetroxide. J. Cell Biol. 12, 385–410. 10.1083/jcb.12.2.385 14483299 PMC2106034

[B42] Pallotto M. Watkins P. V. Fubara B. Singer J. H. Briggman K. L. (2015). Extracellular space preservation aids the connectomic analysis of neural circuits. Elife 4:e08206. 10.7554/eLife.08206 26650352 PMC4764589

[B43] Paulus M. J. Gleason S. S. Kennel S. J. Hunsicker P. R. Johnson D. K. (2000). High resolution X-ray computed tomography: an emerging tool for small animal cancer research. Neoplasia 2, 62–70. 10.1038/sj.neo.7900069 10933069 PMC1531867

[B44] Peddie C. J. Collinson L. M. (2014). Exploring the third dimension: volume electron microscopy comes of age. Micron 61, 9–19. 10.1016/j.micron.2014.01.009 24792442

[B45] Perez A. J. Seyedhosseini M. Deerinck T. J. Bushong E. A. Panda S. Tasdizen T.. (2014). A workflow for the automatic segmentation of organelles in electron microscopy image stacks. Front. Neuroanat. 8:126. 10.3389/fnana.2014.00126 25426032 PMC4224098

[B46] Plaza S. M. Scheffer L. K. Chklovskii D. B. (2014). Toward large-scale connectome reconstructions. Curr. Opin. Neurobiol. 25, 201–210. 10.1016/j.conb.2014.01.019 24598270

[B48] Ramón y Cajal S. (1889). Conexion general de los elementos nerviosos. Med. Pract. 2, 341–346.

[B47] Ramón y Cajal S. (1995). Histology of the Nervous System of Man and Vertebrates. Translated (from the French) by Neely and Larry Swanson. New York, NY: Oxford University Press.

[B49] Rozental R. Giaume C. Spray D. (2000). Gap junctions in the nervous system. Brain Res. Brain Res. Rev. 32, 11–15. 10.1016/s0165-0173(99)00095-8 10928802

[B50] Saalfeld S. Cardona A. Hartenstein V. Tomančák P. (2010). As-rigid-as-possible mosaicking and serial section registration of large ssTEM datasets. Bioinformatics 26, i57–i63. 10.1093/bioinformatics/btq219 20529937 PMC2881403

[B51] Schalek R. Kasthuri N. Hayworth K. Berger D. Tapia J. Morgan J. (2011). Development of high-throughput, high-resolution 3D reconstruction of large-volume biological tissue using automated tape collection ultramicrotomy and scanning electron microscopy. Microsc. Microanal. 17, 966–967. 10.1017/s1431927611005708

[B52] Schleicher A. Amunts K. Geyer S. Morosan P. Zilles K. (1999). Observer-independent method for microstructural parcellation of cerebral cortex: a quantitative approach to cytoarchitectonics. Neuroimage 9, 165–177. 10.1006/nimg.1998.0385 9918738

[B53] Tasdizen T. Koshevoy P. Grimm B. C. Anderson J. R. Jones B. W. Watt C. B.. (2010). Automatic mosaicking and volume assembly for high-throughput serial-section transmission electron microscopy. J. Neurosci. Methods 193, 132–144. 10.1016/j.jneumeth.2010.08.001 20713087 PMC2952705

[B54] Tek F. B. Kroeger T. Mikula S. Hamprecht F. A. (2014). “Automated cell nucleus detection for large-volume electron microscopy of neural tissue” in Biomedical Imaging (ISBI), 2014 IEEE 11th International Symposium on IEEE (Beijing), 69–72. Retrieved from: http://ieeexplore.ieee.org/xpls/abs_all.jsp?arnumber=6867811

[B55] Titze B. Denk W. (2013). Automated in-chamber specimen coating for serial block-face electron microscopy. J. Microsc. 250, 101–110. 10.1111/jmi.12023 23451833

[B56] van Deurs B. (1975). The use of a tannic acid-glutaraldehyde fixative to visualize gap and tight junctions. J. Ultrast. Res. 50, 185–192. 10.1016/s0022-5320(75)80049-9 804041

[B57] Varshney L. R. Chen B. L. Paniagua E. Hall D. H. Chklovskii D. B. (2011). Structural properties of the caenorhabditis elegans neuronal network. PLoS Comput. Biol. 7:e1001066. 10.1371/journal.pcbi.1001066 21304930 PMC3033362

[B58] Wanner A. A. Kirschmann M. A. Genoud C. (2015). Challenges of microtome-based serial block-face scanning electron microscopy in neuroscience. J. Microsc. 259, 137–142. 10.1111/jmi.12244 25907464 PMC4745002

[B100] Wickersham I. R. Finke S. Conzelmann K. K. Callaway E. M. (2006). Retrograde neuronal tracing with a deletion-mutant rabies virus. Nat. Methods 4, 47–49. 10.1038/nmeth999 17179932 PMC2755236

[B59] White J. G. Southgate E. Thomson J. N. Brenner S. (1986). The structure of the nervous system of the nematode Caenorhabditis elegans. Philos. Trans. R. Soc. Lond. B Biol. Sci. 314, 1–340. 10.1098/rstb.1986.0056 22462104

